# MiR-766-3P promotes degenerative lumbar disc disease by regulating SIRT6 to aggravate the inflammatory response of human nucleus pulposus cells

**DOI:** 10.1186/s41065-026-00696-5

**Published:** 2026-06-03

**Authors:** Dongwei An, Pan Yang

**Affiliations:** 1 Orthopedics Department, Yangling Demonstration Zone Hospital, Xianyang, 712100 China; 2 Pain Management Department, Yangling Demonstration Zone Hospital, No. 8, Houji Road, Yangling District, Xianyang, 712100 China

**Keywords:** Lumbar degenerative disc, MiR-766-p, SIRT6, Inflammatory, Extracellular matrix

## Abstract

**Background:**

Lumbar degenerative disc disease (LDD) is a disabling condition which was associated with progressive degeneration of the intervertebral discs. The miR-766-3p and its modulation of SIRT6 remains unclear in LDD.

**Purpose:**

This study aims to explored the regulated function of miR-766-3p and SIRT6 during LDD.

**Methods:**

Human nucleus pulposus cells (HNPCs) were treated with lipopolysaccharide (LPS) to construct a model of LDD to simulate inflammatory conditions. RT-qPCR and ELISA assays were employed to measure the expression of miR-766-3p and SIRT6. Additionally, we evaluated the levels of TNF‑α, IL‑1β, IL‑6, aggrecan, and collagen II by ELISA. Dual-luciferase reporter assays validated direct targeting between miR-766-3p and SIRT6.

**Results:**

We observed that miR-766-3p was upregulated in LDD patients, and SIRT6 was downregulated. It is consistent with the changes in LPS-induced HNPCs. Additionally, when we inhibited the miR-766-3p and overexpressed SIRT6, inflammatory cytokines decreased, whereas aggrecan and collagen II increased. Moreover, the inhibition of miR-766-3p increased SIRT6, which was decreased by LPS. And the binding relationship between miR-766-3p and SIRT6 was confirmed by luciferase activity. MiR-766-3p mimic reversed the effects of elevated SIRT6 expression.

**Conclusion:**

The findings identify the miR-766-p/SIRT6 axis as a critical regulator mediating inflammatory responses and driving extracellular matrix degradation during LDD. Targeting this molecular pathway would offer novel therapeutic strategies for mitigating tissue damage of LDD.

## Introduction

Lumbar degenerative disc (LDD) is a prevalent musculoskeletal disorder that causes chronic lower back pain and functional impairments [1, 2]. The multifactorial mechanisms of LDD included mechanical stress, aging, genetic predisposition, and inflammation-driven catabolic responses within nucleus pulposus cells [3, 4]. These inflammatory responses are one of the reasons for the degradation of key extracellular matrix (ECM) components such as aggrecan and collagen II, which could compromise disc integrity and function [5, 6]. Over recent years, significant advances have promoted the study of the molecular regulators implicated in LDD progression, including microRNAs (miRNAs) [7, 8].

Aberrant expression patterns of specific miRNAs have been implicated in various pathological processes of LDD, including inflammation [9], apoptosis promotion [10], and ECM degradation [11]. Notably, miR-766-3p has obtained attention due to its consistent upregulation in clinical patients with lumbar disc degeneration [7]. It was reported that miR-766-3p promoted apoptosis, inflammation, and oxidative stress in human chondrocytes, which contributed to cartilage degradation [12]. And it damaged osteoarthritic rat models by disrupting ECM homeostasis too [13]. Therefore, upregulation of miR-766-3p may exacerbate joint degeneration by increasing proinflammatory cytokines, which accelerate matrix breakdown.

It is reported that SIRT6 was a crucial factor in bone-related diseases and participated in the onset and progression of osteoarthritis, rheumatoid arthritis, and intervertebral disc degeneration [14]. SIRT6 played a pivotal role in the LDD by modulating inflammatory responses and cellular homeostasis. Evidence showed that overexpression of SIRT6 alleviated these inflammatory effects by promoting autophagy and reducing cell senescence and death [15]. Additionally, DNMT1 can influence macrophage-induced polarization by modulating SIRT6 expression [16]. All the above research predicted SIRT6 as a promising therapeutic target for alleviating inflammation and degeneration in LDD. However, the specific interaction between miR-766-3p and SIRT6 has not been studied clearly. We investigated the regulation and the functional mechanism of both in LDD.

## Materials and methods

### Participants

120 patients undergoing LDD at Yangling Demonstration Zone Hospital were consecutively enrolled in this study, including 60 patients diagnosed with LDD and 60 non-LDD controls who underwent surgery due to trauma. Exclusion criteria for all participants comprised spinal trauma, infection, deformity, prior spinal surgery, autoimmune diseases, malignancies, severe osteoporosis, and hepatic or renal dysfunction.

Before the surgical procedure, blood samples (approximately 5 mL) were collected from all participants. Blood samples were drawn into anticoagulant-treated tubes and processed promptly to isolate serum or plasma, which was subsequently stored at -80℃. This study was approved by the Ethics Committee of Yangling Demonstration Zone Hospital. Written informed consent was obtained from each participant before inclusion in accordance with the Declaration of Helsinki.

### Cell culture and treatment

Primary HNPCs were maintained in DMEM (Merck, Shanghai, China), supplemented with 10% FBS. Cultures were incubated under 5% CO_2_ atmosphere at 37℃. To mimic inflammatory conditions characteristic of LDD, the cells were exposed to 10 ng/mL lipopolysaccharide (LPS; Merck, Shanghai, China) for 0, 6, 12, and 24 h. After this incubation period, the cultures were collected for further experimental evaluation.

### Cell transfection

MiR-766-3p mimics, miR-766-3p inhibitors, and their respective negative controls (mimic NC and inhibitor NC) were obtained from RiboBio. For plasmid-mediated overexpression, SIRT6 overexpression vectors (oe-SIRT6) and corresponding negative control plasmids (NC) were purchased from RiboBio. We used Lipofectamine 2000 reagent (Merck, Shanghai, China) to transfect indicated miRNAs and vectors into NP cells for 48 h.

### RT-qPCR

Extracting Total RNA from cells by TRIzol reagent (Merck, Shanghai, China). We used a PrimeScript RT reagent kit (Takara, Beijing, China) to synthesize Complementary DNA (cDNA). Through using SYBR Green Master Mix (Takara, Beijing, China) for Quantitative PCR. Relative gene expression levels were normalized using the comparative Ct method (2^−ΔΔCt^).

### Cell viability assay

HNPCs (5 × 10^3^/well) were seeded into 96-well plates and allowed to LPS treatment and transfection. To evaluate cell viability, 10 µL of CCK-8 solution (Beyotime, Shanghai, China) was added to each well over a total duration of 24 h. Following incubation with the reagent at 37℃ for 2 h, we measured absorbance values for quantitative assessment at 450 nm on a microplate spectrophotometer.

### Western blot analysis

Protein extraction from HNPC lysates was carried out using RIPA buffer (Sigma) under ice‑cold conditions for 30 min. The resulting mixtures were then centrifuged at 12,000 g for 30 min at 4 ℃ to remove insoluble debris, and protein concentrations were measured with the DC protein assay kit (Bio‑Rad, USA). Equal amounts of each sample were resolved on SDS‑PAGE gels, followed by electrophoretic transfer onto nitrocellulose membranes using the Trans‑Blot Turbo Transfer System (Bio‑Rad). After transfer, membranes were blocked for 1 h in TBST containing 5% non‑fat dry milk. Primary antibody incubation was performed overnight at 4 ℃ with gentle agitation in TBST. The panel of primary antibodies included: TNF-α Antibody (#3707; Cell Signaling Technology, USA), IL-1β monoclonal antibody (#12242; Cell Signaling Technology, USA), IL-6 monoclonal antibody (#12153; Cell Signaling Technology, USA), aggrecan monoclonal antibody (#28971; Cell Signaling Technology, USA), and collagen Ⅱ monoclonal antibody (#43306; Cell Signaling Technology, USA). After washing, HRP‑conjugated secondary antibodies against mouse and rabbit (1:10,000, Abcam) were applied. Chemiluminescent signals were visualized using a ChemiDoc system (Bio‑Rad, USA), and band intensities were quantified with Quantity One software (Bio‑Rad, USA).

### ELISA assay

Cell culture lysates and serum were collected from each treatment group for analysis. Specific ELISA kits targeting TNF-α, IL-1β, IL-6 (Abcam, Shanghai, China), Collagen II, and Aggrecan (Beyotime, Shanghai, China) were used. Measurements of absorbance at 450 nm were conducted using a microplate spectrophotometer.

### Dual-luciferase reporter assay

Potential targets of miR-766-3p were predicted using the TargetScan database. Plasmid constructs of the wild-type (wt-SIRT6) and mutated (mut-SIRT6) were generated. The cells were co-transfected with vectors along with miR-766-3p mimics, inhibitors, or respective negative controls using Lipofectamine 2000 reagent. Luciferase activity was assessed 48 h post-transfection utilizing the Dual-Luciferase Reporter Assay Kit (Beyotime, Shanghai, China) to evaluate the direct targeting relationship.

### Statistical analysis

Data are presented as mean ± standard deviation. Statistical analyses were performed using Graphpad 10.1.2. For comparisons of multiple groups, one-way analysis of variance (ANOVA) was applied. Differences between two groups were assessed by an unpaired Student’s t-test for normally distributed data or the Mann-Whitney U test for nonparametric data. And *p* < 0.05 was considered statistically significant throughout the study.

## Results

### The levels of miR-766-3p and SIRT6 in patients with LDD

**Fig. 1:**
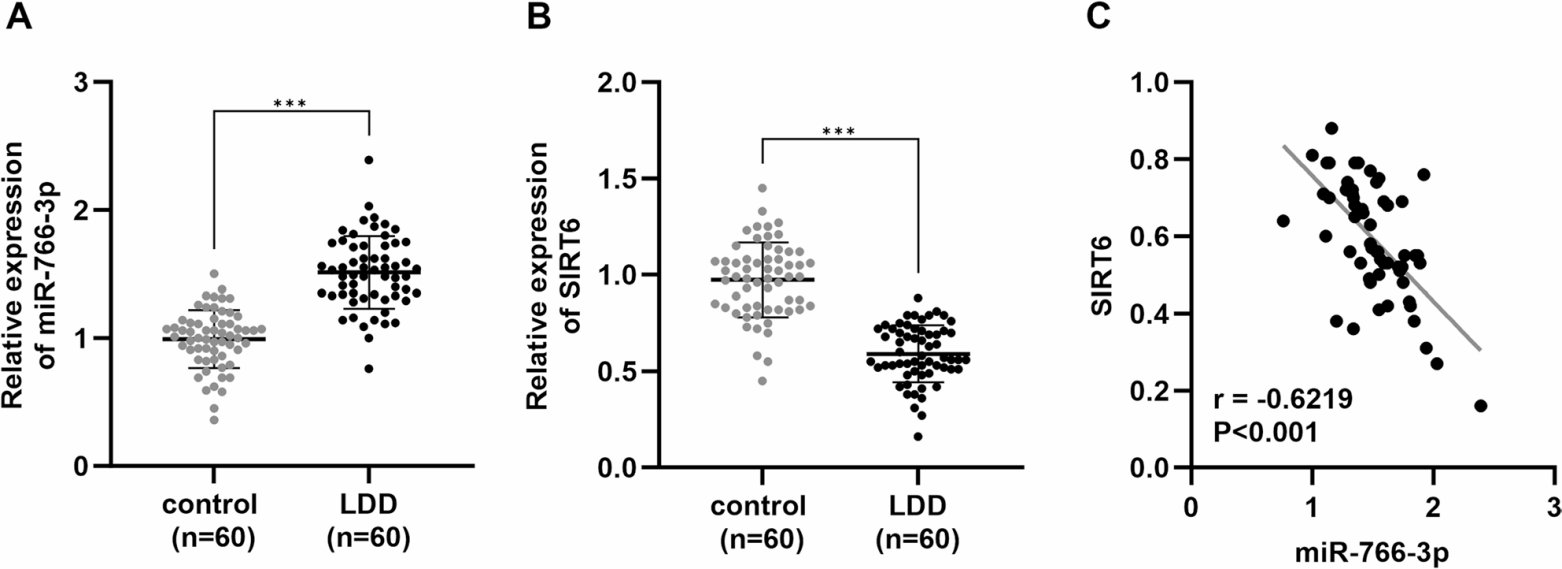
Expression of miR-766-3p and SIRT6 in patients with LDD. **A**: MiR-766-3p was upregulated in LDD; **B**: SIRT6 was downregulated in LDD; **C**: Negative correlation between miR-766-3p and SIRT6

The expression of miR-766-3p and SIRT6 in patients with LDD was analyzed to elucidate their potential roles in LDD. MiR-766-3p levels were significantly upregulated (Fig. 1A). Conversely, SIRT6 expression was markedly decreased (Fig. 1B). Furthermore, correlation analysis revealed a negative association (*r* = -0.6219) between SIRT6 and miR-766-3p (Fig. 1C). We think miR-766-3p may have regulatory effects on SIRT6.

### Effect of LPS induction on HNPCs

**Fig. 2:**
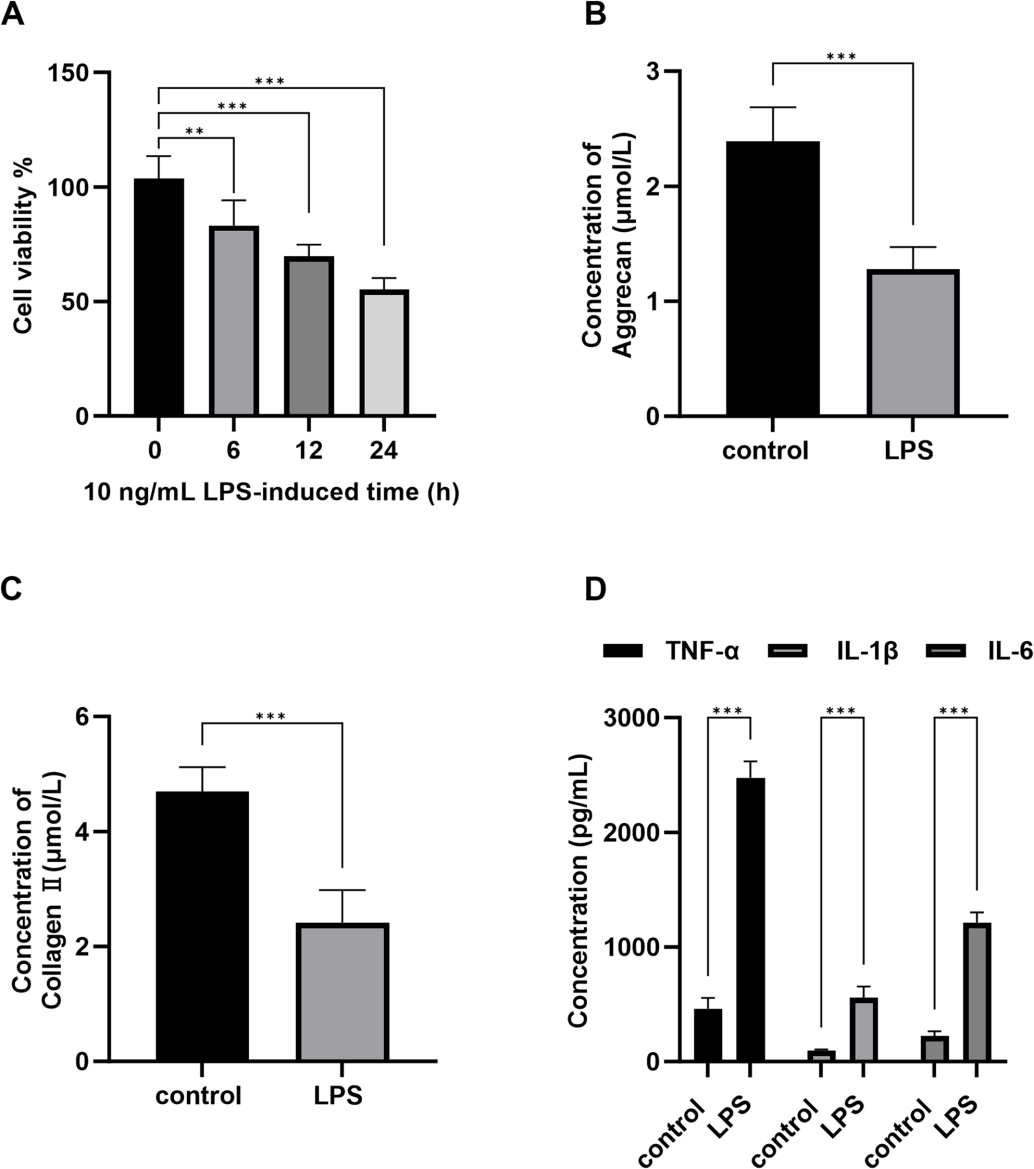
Effect of LPS on HNPCs. **A**: Cell viability declined with the LPS-induced time; **B-C**: The concentration of aggrecan and collagen Ⅱ decreased in LPS-induced cells; **D**: The concentration of pro-inflammatory cytokines was reduced in LPS-induced cells

The effects of LPS stimulation on HNPCs were investigated to establish an in vitro inflammation model of LDD. As illustrated in Fig. 2A, cellular viability exhibited a time-dependent decline following LPS exposure time. Concurrently, significant reductions in the concentrations of Aggrecan and Collagen II (Fig. 2B and C) were observed in LPS-induced cells. Moreover, TNF-α, IL-1β, and IL-6 were elevated in response to LPS stimulation (Fig. 2D), confirming activation of an inflammatory cascade within HNPCs. Collectively, these results validated the success of the LPS-induced HNPCs model for studying inflammation in LDD.

### MiR-766-3p reversed the effects induced by LPS in the cell model

**Fig. 3:**
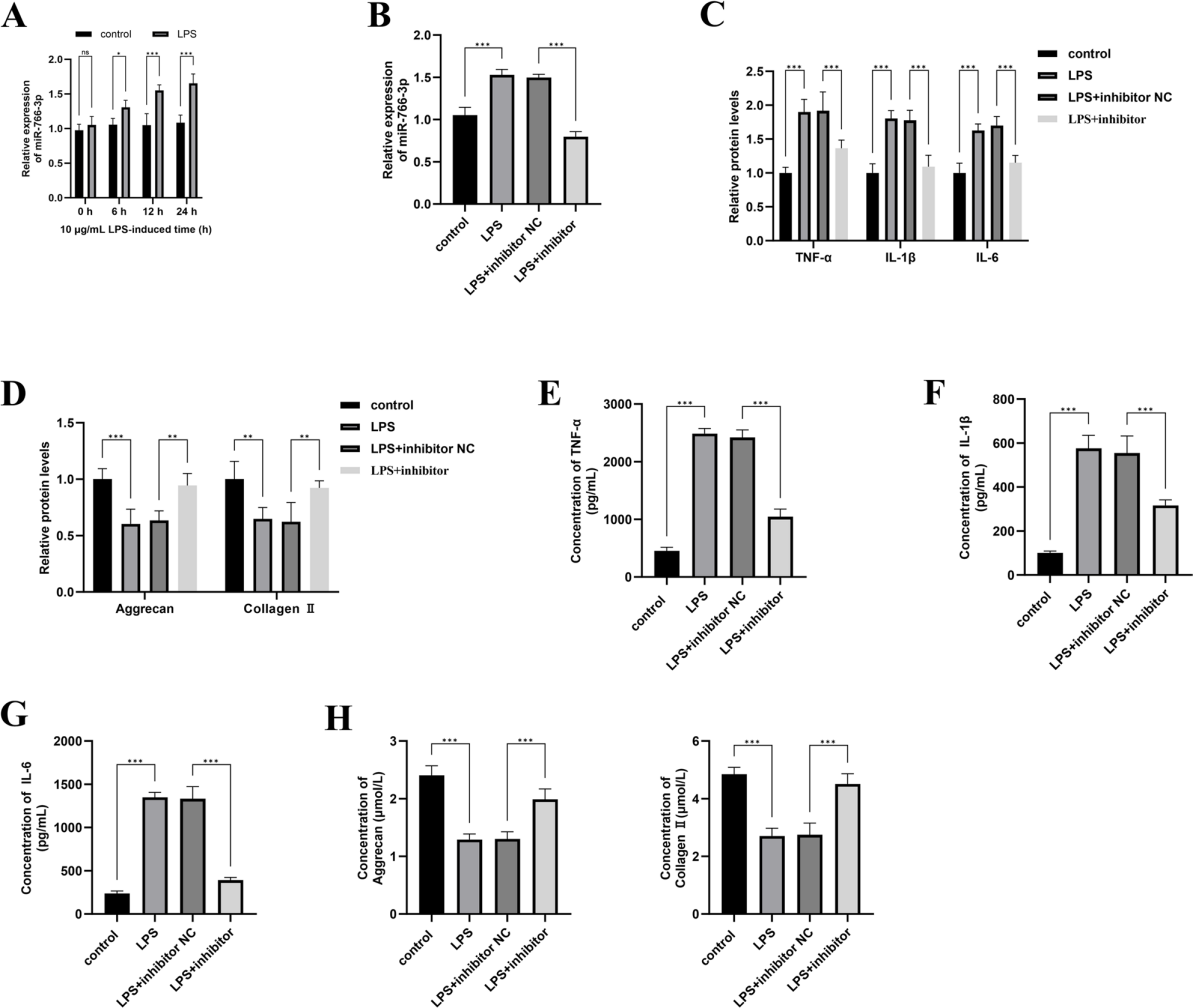
Effects of miR-766-3p on LPS-induced HNPCs. **A**: MiR-766-3p increased with the LPS-induced time; **B**: High transfection efficiency of miR-766-3p inhibitor in LPS-induced cells; **C**: Protein quantification of intracellular TNF-α, IL-1β, and IL-6 using Western blots. **D**: Protein quantification of intracellular Aggrecan and Collagen II using Western blots. **E-G**: MiR-766-3p inhibitor reduced the level of pro-inflammatory cytokines in LPS-induced cells; **H**: MiR-766-3p inhibitor increased the concentration of aggrecan and collagen Ⅱ

The regulated function of miR-766-3p was evaluated in the LPS-induced cell model. In Fig. 3A, miR-766-3p expression progressively increased with prolonged LPS treatment. We confirmed the efficient transfection of miR-766-3p inhibitor into LPS-induced cells by successful regulation of miR-766-3p (Fig. 3B). Western blot analysis showed that protein levels of TNF-α, IL-1β, and IL-6 were increased in LPS-treated cells compared to controls, while miR-766-3p inhibitor decreased the protein levels of TNF-α, IL-1β, and IL-6 compared with negative controls (Fig. 3C). Then, western blotting was applied to estimate the protein expression of ECM-related markers (Collagen II and Aggrecan) in cells and found that LPS treatment obviously suppressed the expression of collagen II and aggrecan; however, inhibition of miR-766-3p augmented the expression of Collagen II and Aggrecan in HNPCs (Fig. 3D). Functional experiments were used to reveal the effects that the inhibition of miR-766-3p could suppress the levels of pro-inflammatory cytokines, which were elevated by LPS-induced (Figs. 3E-G), suggesting the importance of miR-766-3p in mediating inflammatory responses. Furthermore, the LPS downregulated concentrations of aggrecan and collagen II were reversed through inhibiting miR-766-3p, indicating it restored ECM integrity (Figs. 3H). These findings highlight the critical function of miR-766-3p in promoting inflammation and matrix degradation within LPS-induced HNPCs.

### Confirmation of regulatory relationship between miR-766-3p and SIRT6

**Fig. 4:**
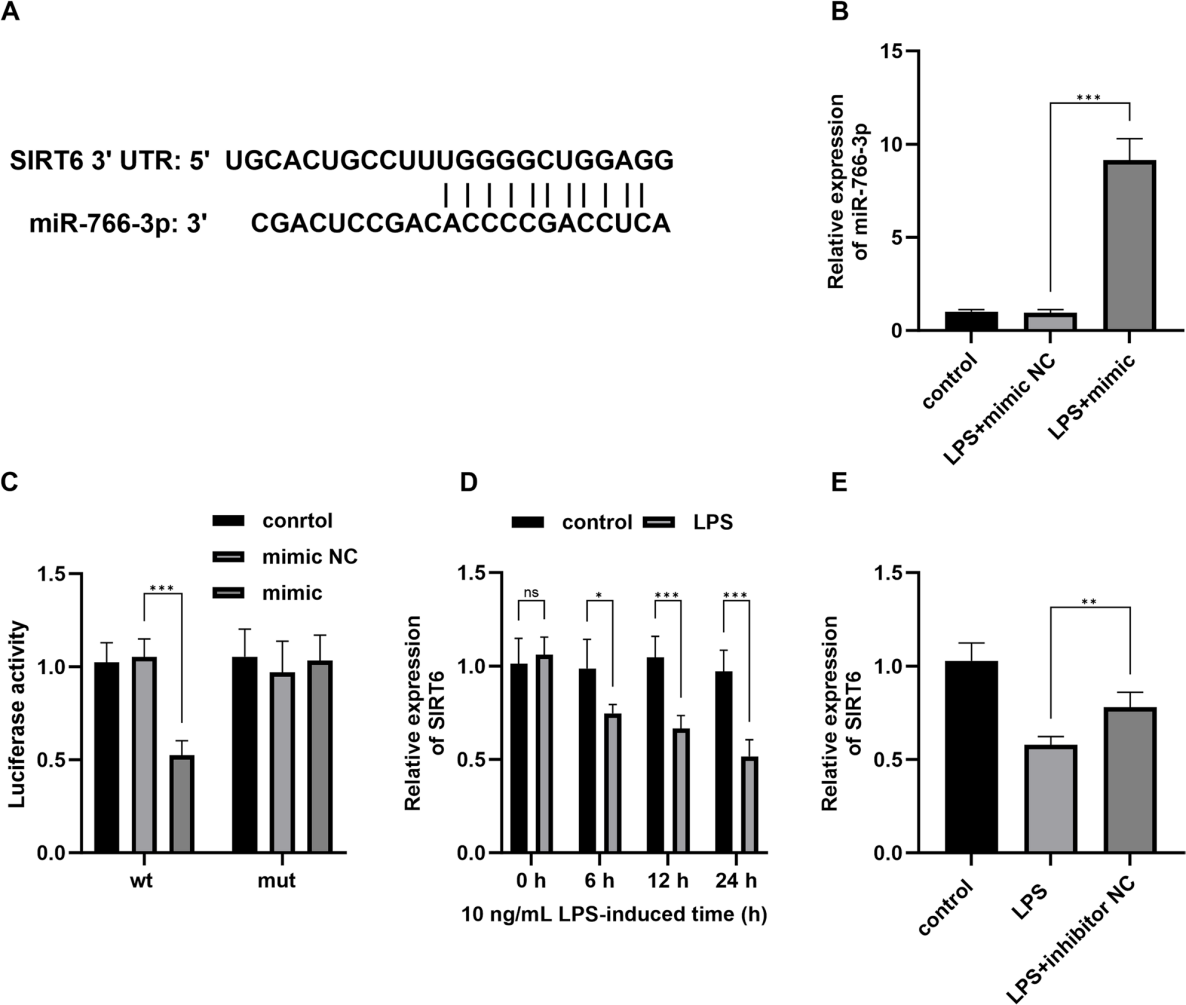
Confirmation of the binding relationship between miR-766-3p and SIRT6. **A**: The predicted binding site; **B**: High transfection efficiency of miR-766-3p mimic; **C**: Luciferase activity assay; **D**: The level of SIRT6 declined with LPS-induced time; **E**: The level of SIRT6 was upregulated by miR-766-3p inhibitor

Through molecular and cellular experiments, the negative-regulated relationship of miR-766-3p to SIRT6 was validated. Initially, the predicted binding sites of miR-766-3p and SIRT6 were identified in the TargetScan database (Fig. 4A). Successful transfection efficiency of miR-766-3p mimics into LPS-induced cells was confirmed (Fig. 4B). Functionally, luciferase reporter assays showed a decrease in luciferase activity by co-transfection with miR-766-3p mimic and wild-type SIRT6 3’-UTR constructs (Fig. 4C). The results confirmed that SIRT6 was specifically targeted by miR-766-3p. In parallel, SIRT6 expression progressively declined with increasing durations of LPS exposure (Fig. 4D). Importantly, the decrease of SIRT6 levels was restored after inhibiting miR-766-3p in LPS-induced cells (Fig. 4E), which indicated that miR-766-3p negatively regulated SIRT6 expression. Collectively, these data firmly establish that miR-766-3p directly targets and suppresses SIRT6 under inflammatory conditions associated with LDD.

### Expression of miR-766-3 reverses the effects of overexpressing SIRT6

**Fig. 5:**
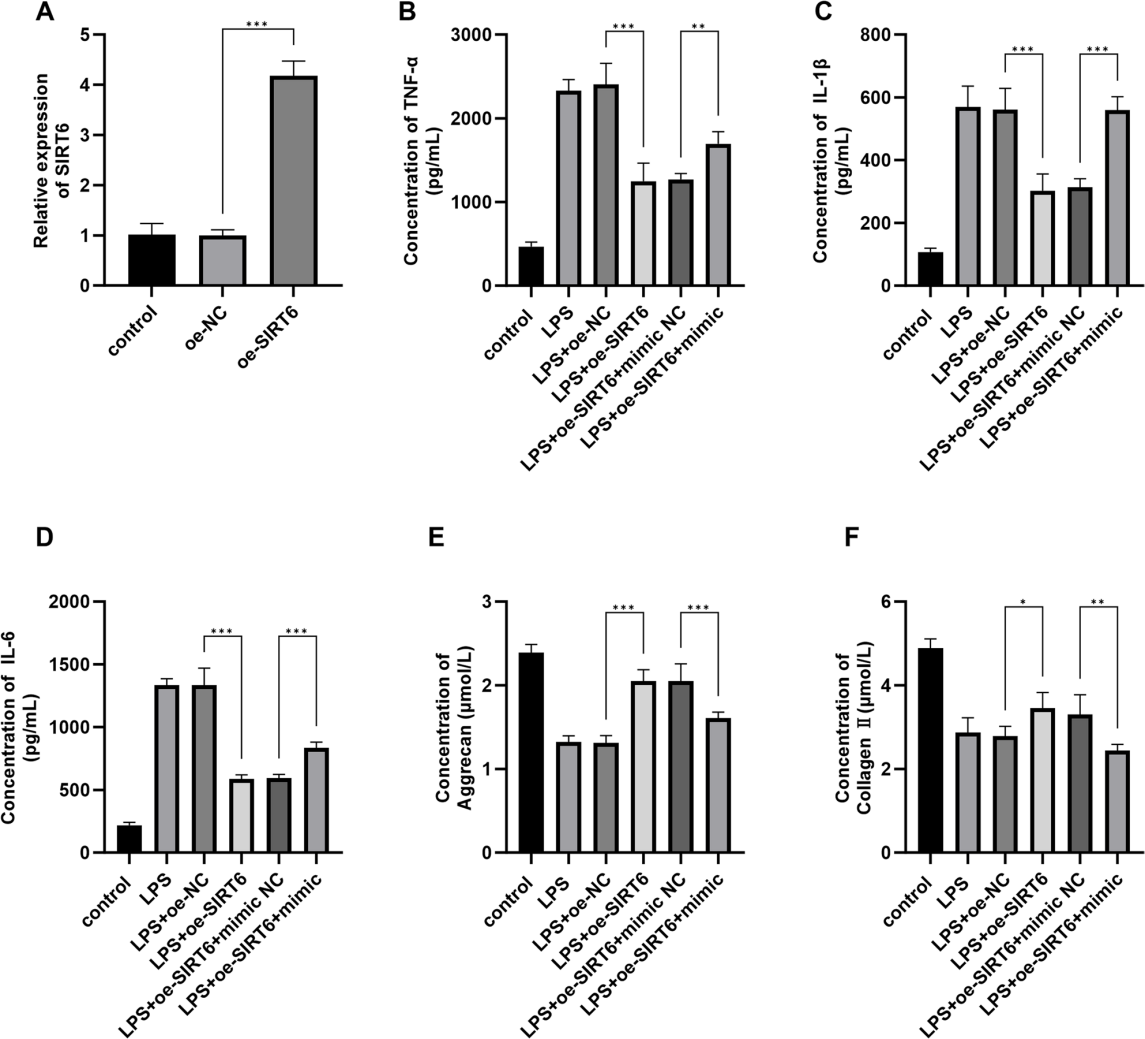
Overexpression of miR-766-3 reversed the effect of overexpressing SIRT6. **A**: High transfection efficiency of oe-SIRT6; **B-D**: MiR-766-3 mimic reversed the decreased pro-inflammatory cytokines caused by overexpressing SIRT6; **E-F**: MiR-766-3 mimic reversed the increased aggrecan and collagen Ⅱ caused by overexpressing SIRT6

The interaction between miR-766-3p and SIRT6 in regulating inflammatory responses was investigated. As shown in Fig. 5A, successful transfection of the SIRT6 overexpression plasmid (oe-SIRT6) was confirmed, establishing a reliable model for subsequent analyses. Overexpression of SIRT6 significantly decreased LPS-induced elevation of TNF-α, IL-1β, and IL-6 levels. However, overexpressing miR-766-3p effectively reversed the protective influences of SIRT6, restoring elevated pro-inflammatory cytokine expression (Figs. 5B-D). Furthermore, the increased concentrations of aggrecan and collagen II by overexpressing SIRT6 on ECM degradation were reversed by miR-766-3p upregulation (Figs. 5E-F). This reversal highlights that miR-766-3p prevents the anti-inflammatory function of SIRT6 overexpression by downregulating it.

## Discussion

LDD is one of the reasons for chronic low back pain [17]. Inflammation and ECM degradation play critical roles in the pathogenesis of LDD. Recent studies have shown that inflammatory mediators such as TNF-α, IL-1β, and reactive oxygen species promote ECM breakdown by upregulating matrix metalloproteinases while downregulating their inhibitors, accelerating disc degeneration [18, 19]. Moreover, cellular stress response proteins regulate key pathways, like SQSTM1/p62 regulated NF-κB activation and oxidative stress to inflammation-induced ECM remodeling and cell death within the intervertebral disc [20]. Additionally, miRNA regulation also impacts inflammatory signaling and ECM metabolism [21–23]. MiR-340-5p was found to protect nucleus pulposus cells from LPS-induced damage through modulation of SOX4 expression, highlighting potential therapeutic targets for LDD treatment [24]. So intertwined nature of inflammation and ECM degradation crucially drives LDD progression.

In recent years, miR‑766‑3p has emerged as an important modulator in various pathological contexts, including cancer progression and inflammatory diseases [25, 26]. And these results were also identified in our study, that miR-766-3p was markedly upregulated in LDD patient samples. The current work extends how overexpression of miR‑766‑3p aggravates LDD. MiR-766-3p increased the pro-inflammatory cytokines and the degradation of aggrecan and collagen II in LPS-induced HNPCs. We predicted that miR-766-3p upregulation contributed to ECM degradation and inflammation for damaging the cellular homeostasis of HPNCs. Disruption of cell homeostasis in HNPCs will cause cell aging and apoptosis, which promotes the LDD.

SIRT6 plays protective roles via inhibition of reactive oxygen species generation and modulation of glucose metabolism pathways relevant to cell survival under stress conditions [27, 28]. Previous studies have shown that the reduction of SIRT6 exacerbates intervertebral disc degeneration via disrupted cellular senescence [29]. SIRT6 has emerged as a protective factor of inflammatory responses and cellular aging [15, 30]. Consistent with the previous study, we demonstrate that SIRT6 is concurrently downregulated. Our correlation data and luciferase activity assay reveal the inverse regulatory relationship between miR-766-3p and SIRT. At the same time, our functional assays demonstrated that overexpressed SIRT6 suppressed LPS-induced inflammation effectively, whereas upregulation of miR-766-3p reversed this protection. It elucidated the regulatory role of miR-766-3p on SIRT6 expression in LDD and verified the significance in modulating inflammatory responses and ECM homeostasis.

Nevertheless, the HNPCs were employed to model inflammation-induced degeneration, but the animal models remain necessary for comprehensive validation in future studies. In a word, this research reveals that miR-766-3p is significantly upregulated in LDD and negatively regulates the expression of SIRT6. And inhibiting miR-766-3p alleviated inflammatory responses and preserved ECM by increasing SIRT6 levels. Our findings elucidated the critical molecular axis miR-766-3p/SIRT6 axis in driving LDD progression and provided a promising therapeutic target for treating LDD.

## Data Availability

Corresponding authors may provide data and materials.
